# A review of material design for high performance triboelectric nanogenerators: performance improvement based on charge generation and charge loss

**DOI:** 10.1039/d4na00340c

**Published:** 2024-07-24

**Authors:** Xiaochuan Li, Qianxi Yang, Dahu Ren, Qianying Li, Huake Yang, Xuemei Zhang, Yi Xi

**Affiliations:** a Chongqing Key Laboratory of Soft Condensed Matter Physics and Smart Materials, Department of Applied Physics, Analytical and Testing Center, Chongqing University Chongqing 400044 P. R. China yxi6@cqu.edu.cn

## Abstract

As a type of innovative device, triboelectric nanogenerators (TENGs) are capable of converting mechanical energy into electrical energy through the triboelectric effect. Based on the working mechanism, the output performance of TENGs heavily relies on the triboelectric materials used. The modification of triboelectric materials is the most efficient way to improve the output performance of TENGs. Herein, this review focuses on the recent progress in triboelectric material design for high-performance TENGs. First, the basic theory of TENGs is introduced. Second, the relationship between the triboelectric materials and the output performance of TENGs is summarized in detail based on a theoretical model of the triboelectric charge dynamic equilibrium. Furthermore, the relevant strategies are analyzed in detail. Finally, challenges and shortcomings of the triboelectric materials for high-performance TENGs are discussed. This review provides a basis for the research status and future development of triboelectric materials.

## Abstract 2

This review summarizes the relevant material-design strategies for improving the output performance of TENGs based on charge generation and charge loss.

## Graphical abstract

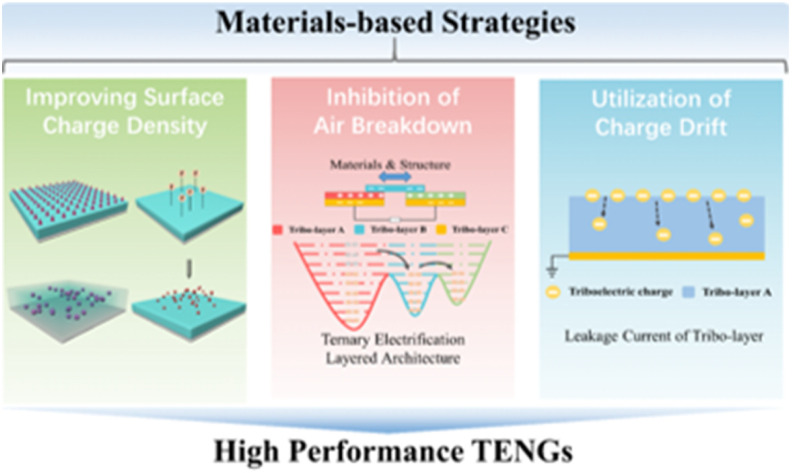

## 1 Introduction

Since the beginning of the 21st century, the increasing demand for energy in human society has led to the extensive use of fossil fuels such as coal, oil, and natural gas, resulting in increasingly serious carbon emissions and environmental issues.^1,2^ In recent years, the use of renewable energy, including solar energy, biological energy, ocean energy, thermal energy, wind energy, and chemical energy, has gradually increased its share in society due to its advantages of low carbon emissions and minimal environmental pollution.^3–5^ Various energy-harvesting devices such as electromagnetic generators (EMGs), solar cells,^6,7^ thermoelectric generators,^8,9^ piezoelectric nanogenerators (PENGs),^10–12^ and triboelectric nanogenerators (TENGs)^13–16^ have been designed for the production of different types of renewable energy. Based on the coupling effect of triboelectrification and electrostatic induction, TENGs were first invented by Wang's group in 2012 and have attracted increasing attention among the above-mentioned devices for harvesting energy due to its simple design, high voltage output, low cost, wide material selection, and high energy conversion efficiency at low frequencies.^17–21^ Their seminal work laid the foundation for the further exploration of TENGs. Since its invention, TENGs have developed rapidly with contributions from researchers all over the world and have become an idea for energy harvesting technology to be applied in various applications such as micro/nano power sources, self-powered sensing, blue energy harvesting, and high-voltage power sources.^22–33^ Despite significant progress, the practical application of TENGs is seriously hindered by its low output power and energy conversion efficiencies.

Over the years, the improved output performance of TENGs has been significantly propelled by advancements in mechanical structure design and power management.^34–38^ However, the output performance of TENGs greatly depends on the dynamic equilibrium of triboelectric charge generation and charge loss; thus, simply increasing the triboelectric charge generation is not sufficient to further improve the output performance of TENGs.^39^ Triboelectric materials play a crucial role in determining the output performance of TENGs as it directly influences triboelectric charge generation and charge loss.^40^ Therefore, it is pivotal to study the working mechanisms of triboelectric materials in charge generation and charge loss to further improve the energy harvesting efficiency and expand the application range of TENGs.

**Fig. 1:**
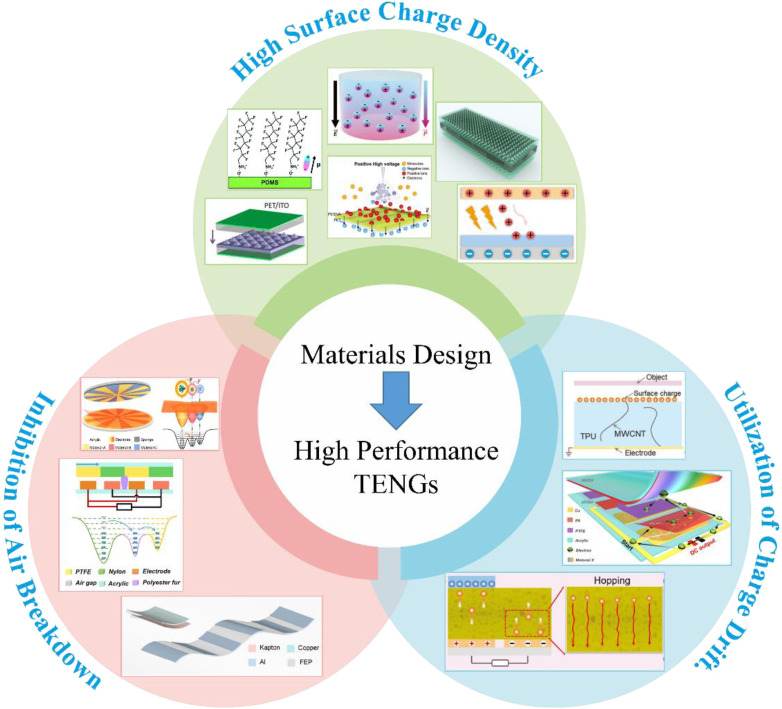
Schematic overview of the strategies to improve the output performance of TENGs. Reprinted with permission from ref. 18. Copyright 2012, American Chemical Society. Reprinted with permission from ref. 41. Copyright 2021, Royal Society of Chemistry. Reprinted with permission from ref. 42. Copyright 2020, Wiley. Reprinted with permission from ref. 43. Copyright 2023, Wiley. Reprinted with permission from ref. 44. Copyright 2020, American Chemical Society. Reprinted with permission from ref. 45. Copyright 2021, Elsevier. Reprinted with permission from ref. 46. Copyright 2023, Wiley. Reprinted with permission from ref. 47. Copyright 2024, Wiley. Reprinted with permission from ref. 48. Copyright 2023, Royal Society of Chemistry. Reprinted with permission from ref. 49. Copyright 2022, Elsevier. Reprinted with permission from ref. 50. Copyright 2022, Wiley. Reprinted with permission from ref. 51. Copyright 2023, Elsevier.

Here, various material design strategies that improve the output performance of TENGs will be introduced accordingly, as shown in Fig. 1. First, the basic working modes of TENGs and the basic theory of TENGs are briefly introduced. Second, the theoretical model of triboelectric charge dynamic equilibrium is described, and the relationship between the triboelectric materials and the TENGs' output performance is summarized. Then, the relevant material-design strategies for improving the output performance of TENGs are described in detail, including surface engineering, inhibition of air breakdown, and utilization of charge drift. Finally, the current challenges in augmenting the output performance of TENGs are discussed, and a brief perspective on future opportunities is also provided.

## 2 Basic working principle of TENGs

### 2.1 Basic working modes of TENGs

**Fig. 2:**
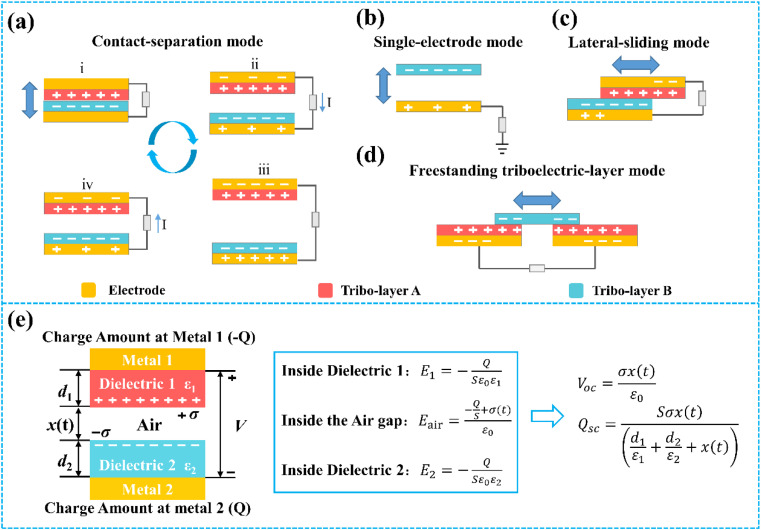
Four basic working modes of TENGs: (a) vertical CS mode, (b) SE mode, (c) LS mode, (d) FT mode. (e) Schematic diagram showing the working principle of the vertical CS mode.

TENGs typically generate transferred charge through the physical contacts between two different types of materials.^52–54^ Such transfer charge between two materials can be attributed to the interaction of their electron clouds.^55,56^ TENGs have four basic working modes: vertical contact-separation (CS) mode, lateral-sliding (LS) mode, single-electrode (SE) mode, and freestanding triboelectric-layer (FT) mode.^40,57–59^ The working principle of the vertical CS-mode TENGs is shown in Fig. 2a. When two different types of triboelectric materials come into contact, opposite triboelectric charges are generated on the surface of the dielectric material due to contact electrification (Fig. 2a(i)). When the two materials are separated by an external mechanical force, the triboelectric charges generated by contact electrification also separate and create an induced potential difference on the electrodes of the corresponding material, thereby causing a current pulse in the external circuit (Fig. 2a(ii)). When the two materials are subjected to separation and contact under an external force, the potential difference between the electrodes disappears, the electrons flow back to the original electrode, and a reverse current pulse occurs in the external circuit (Fig. 2a(iv)). By repeating this contact-separation process, TENGs can continuously generate alternating current output in the external circuit.

The other three working modes are shown in Fig. 2b–d. In the case of SE mode (Fig. 2b), when the triboelectric material approaches or leaves the electrode, charges will transfer from the electrode to the tribo-layer with high electron-withdrawing ability. As a result, an induction current is created in the electrode to balance the electrical potential. This mode has only one electrode and allows the triboelectric materials to move freely, which simplifies the device structure and makes integration with other electronic devices or systems easier.^60–63^Fig. 2c shows a schematic diagram of the LS mode TENGs. The LS mode TENGs are based on the contact sliding between two tribo-layers. When the two tribo-layers slide against each other under an external force, the charges on the tribo-layers also separate and create a potential difference between the two electrodes. When the external circuit is connected, electrons will flow from one electrode to the other due to the existence of a potential difference, forming an electric current. Therefore, the LS mode TENGs are able to collect many forms of mechanical energy, such as human movement and mechanical vibration, and convert it into electrical energy for driving small electronic devices.^64^ The schematic diagram of the FT mode TENGs is shown in Fig. 2d. The reciprocating slide of the independent tribo-layer on two stationary tribo-layers will cause a potential difference between two bottom electrodes, which drives electrons to flow back and forth between the two electrodes through an external circuit load. In this mode, the triboelectric charge can remain on the surface of the tribo-layer for a longer period, which enables stable output and high energy conversion efficiency.^65,66^

### 2.2 Basic theory of TENGs

The theoretical source of TENGs comes from Maxwell's displacement current. In general, Maxwell's displacement current can be defined as follows:1
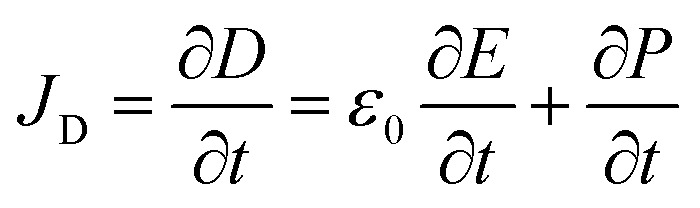
where *D* is the displacement field, *E* is the electric field, *P* is the electric field, and *ε*_0_ is the vacuum permittivity. However, in the case of TENGs, triboelectric charges generated by the physical contact between two different materials also contribute to the displacement current.^67^ Therefore, to account for the influence made by triboelectric charges, Wang *et al.* added an additional polarization density term *P*_S_ in *D* and extended Maxwell's equations.^68,69^ The specific Maxwell's displacement current in TENGs is as follows:2
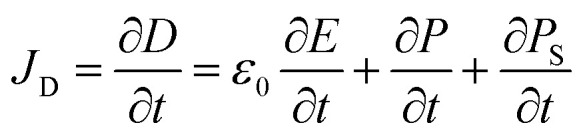


The first two terms on the right side of eqn (2) are induced currents generated by changing electric fields, which is the theoretical basis of the electromagnetic wave existence.^70,71^ The final term is the current caused by the polarization field generated by the electrostatic charge on the surface, which is the theoretical source of TENGs.^20^

To predict the output behavior of TENGs, researchers have proposed several theoretical models.^72–75^ Here, as the CS mode is the most commonly designed, taking the CS mode as an example, the basic output parameter of TENGs can be deduced by a planar plane capacitance model (Fig. 2e).^73^ As shown in Fig. 2e, the vertical CS mode normally consists of two electrodes and two tribo-layers. The two tribo-layers have thicknesses of *d*_1_ and *d*_2_ and dielectric constants of *ε*_1_ and *ε*_2_, respectively. The distance between two tribo-layers is defined as *x*(*t*) and changed by the external mechanical force. When the two tribo-layers are contacted by an external force, the inner surface of the two tribo-layers will produce the same amount of positive and negative charge, respectively. During the separation of the two tribo-layers, an air gap is formed between the two tribo-layers, and the induced potential (*V*) between the two electrodes is generated. The transferred charge between metal 1 and metal 2 is *Q*. By using Gauss's theorem, the induced potential (*V*) between the two electrodes can be given by:^73^3
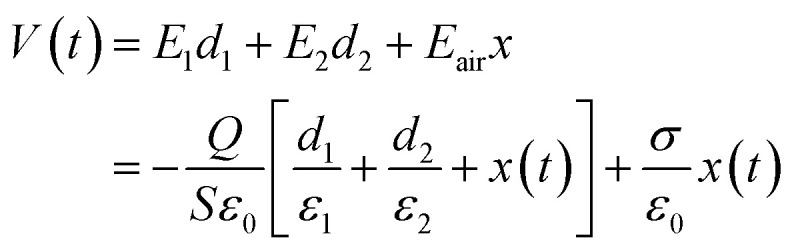
where *ε*_0_ is the vacuum permittivity, and *E*_1_, *E*_2_, and *E*_air_ are the electric field intensity inside dielectric 1, dielectric 2, and the air gap, respectively. From eqn (3), at an open-circuit condition, there is no charge transfer, so *Q* = 0. Therefore, the open-circuit voltage (*V*_OC_) is given by:^73^4
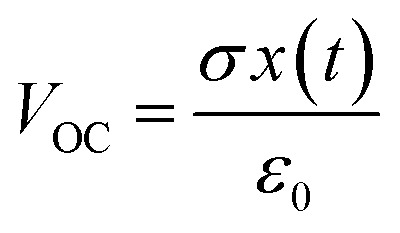


In the short-circuit condition, *V* = 0. Therefore, the transferred charges (*Q*_SC_) and short circuit current (*I*_SC_) are:^73^5
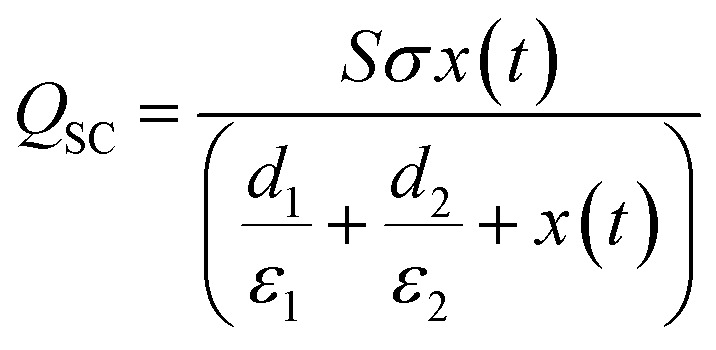
6
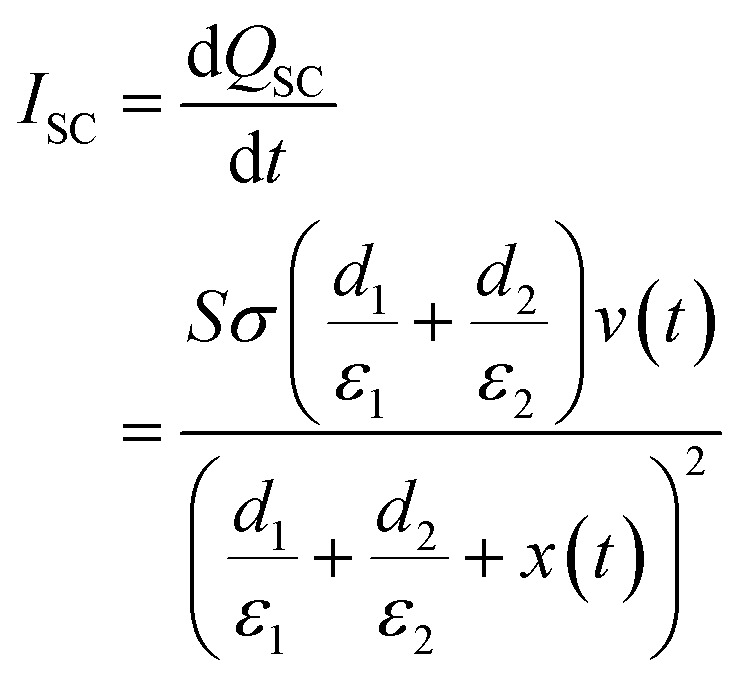


## 3 The relationship between the TENG's output performance and triboelectric materials

**Fig. 3:**
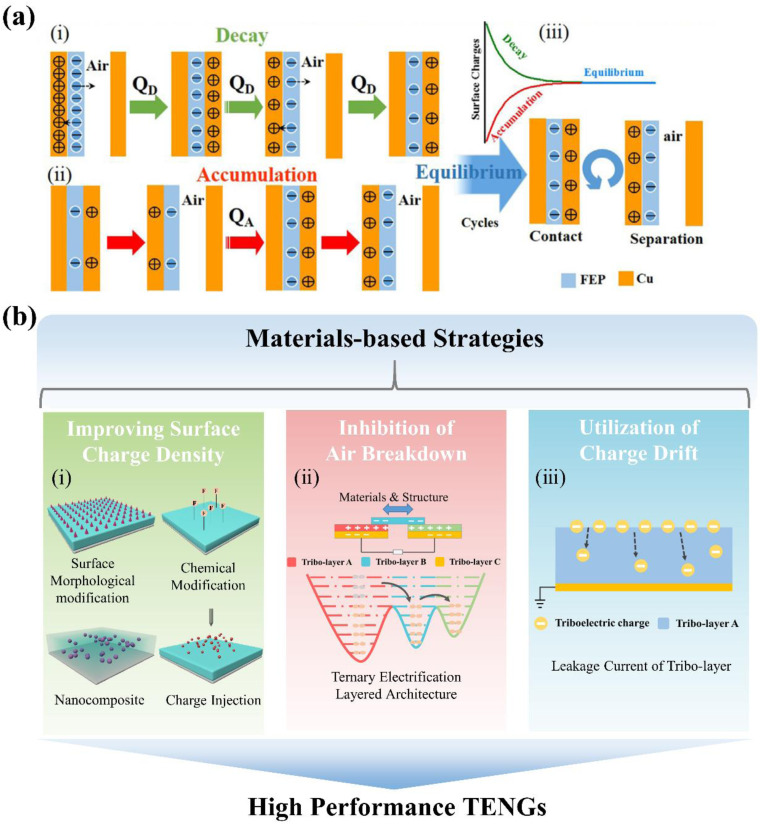
(a) Theory model of the triboelectric charge in dynamic equilibrium. Reprinted with permission from ref. 39. Copyright 2020, Elsevier. (b) Various materials-related strategies to improve the performance of TENGs.

In the actual testing process, the TENGs achieve a maximum output performance only when the triboelectric charge generation and charge loss reach a dynamic equilibrium. In order to further improve the output of TENGs, a theoretical model about the dynamic equilibrium was proposed by Zhang *et al.*, as shown in Fig. 3a.^39^ In this model, the triboelectric charge generated on the surface of the triboelectric materials can be moved or stored within the materials to accumulate the charge and then improve the output performance of TENGs. Additionally, the triboelectric charges can diffuse into the atmosphere or transfer to the bottom electrode, which then influences the output performance. Therefore, the key factor limiting the maximum TENGs' output performance is not only the triboelectric charge generation, but also the triboelectric charge loss.

Charge generation is closely related to the electron withdrawing/donating abilities of triboelectric materials. Among these, the electron withdrawing/donating abilities of triboelectric materials mainly depends on their chemical structure. Many studies have proposed different physical properties to explain the connection between the electron withdrawing/donating ability and chemical structure.^76–80^ However, only a limited number of polymers adhere to these correlations. In 2019, Zou *et al.* built a standardized quantified triboelectric series by summarizing the measurement results of over 50 triboelectric materials.^81^ The triboelectric series can help researchers choose the best material pairs for high performance more carefully. Furthermore, the contribution of functional groups on triboelectric materials to the contact electrification effect is also recognized and proposed.^82^ In summary, there are various ways to modify the triboelectric properties of triboelectric materials: surface morphological modification, chemical modification, nanocomposite, and charge injection (Fig. 3b(i)).

Charge loss can be divided into two subprocesses: air breakdown and charge drift.^40,83^ Furthermore, air breakdown that occurs between the surface of triboelectric materials and the atmosphere has also been identified as another factor restricting the output performance of TENGs.^84–86^ For air breakdown, it is important to select the appropriate material and structure design to reduce the voltage and limit the air breakdown, thus improving the output performance of TENGs, as shown in Fig. 3b(ii). In addition to air breakdown, triboelectric charge drift is also a major mode of triboelectric charge loss. Fig. 3b(iii) presents the strategy for the utilization of charge drift. During the working process of TENGs, the triboelectric charges generated on the surface of materials can transfer to the bottom electrode and achieve the utilization of the transferred charges, thus enhancing the TENGs' output performance.^46,87^

In summary, the process of contact electrification can be divided into three steps: triboelectric charge generation, charge storage, and charge loss. All these steps are mainly related to the triboelectric materials. TENGs can retain a high output performance by improving the surface charge density, inhibition of air breakdown, and utilization of charge drift through triboelectric material design.

## 4 Material design for improving surface charge density

### 4.1 Surface morphological modification

Surface morphological modification is the most commonly used method to improve the output of TENGs, which primarily involves building micro/nanostructures on the tribo-layer surface. The micro/nanostructures can efficiently improve the output by increasing the contact area.

**Fig. 4:**
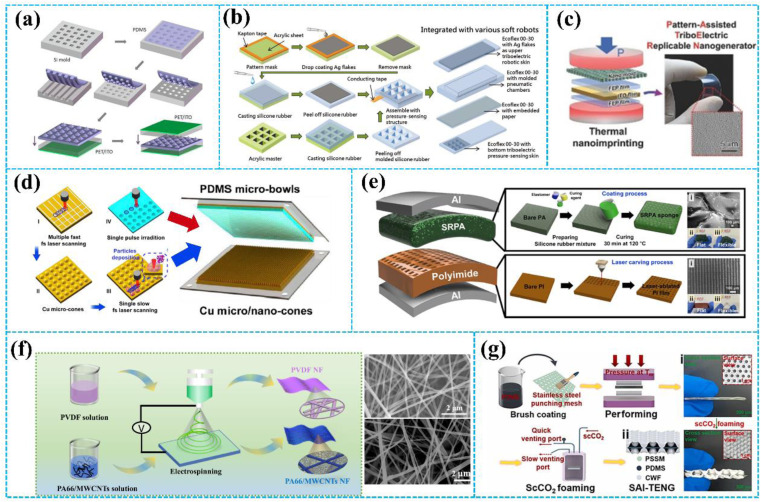
(a) The fabrication process of the flexible TENG. Reprinted with permission from ref. 18. Copyright 2012, American Chemical Society. (b) The process of fabrication of the tribo-layer with triangular micro-prisms structure. Reprinted with permission from ref. 88. Copyright 2018, Wiley. (c) One-step fabrication process of the nano-PATERN using a thermal nanoimprint process. Reprinted with permission from ref. 89. Copyright 2015, Wiley. (d) Preparing micro/nano structures by femtosecond laser direct writing. Reprinted with permission from ref. 90. Copyright 2019, Elsevier. (e) Fabrication procedure of a TENG with a coupled structure using a direct UV laser-ablated film and soft composite of the sponge layer. Reprinted with permission from ref. 91. Copyright 2021, American Chemical Society. (f) Schematic preparation process of TENG incorporating electrospun PVDF and PA66/MWCNTs nanowires. Reprinted with permission from ref. 92. Copyright 2021, Elsevier. (g) The fabrication process of TENG by scCO_2_ foaming. Reprinted with permission from ref. 93. Copyright 2023, Elsevier.

Owing to its simple and low-cost production process, the template method has been widely used to construct micro/nanostructures on the tribo-layer surface. Fig. 4a shows TENGs with three types of regular and uniform polymer patterned arrays (line, cube, and pyramid) prepared by the template method,^18^ which improve the output performance of the TENGs by increasing the triboelectric effect and the capacitance change. Similarly, in Fig. 4b, Lai *et al.* proposed a stretchable and compliant triboelectric robotic skin with triangular micro-prism structure tribo-layer.^88^ The tribo-layer with triangular micro-prism surfaces simultaneously possesses excellent stretchability and excellent sensitivity in low-pressure regimes. Additionally, Choi *et al.* fabricated a pattern assisted triboelectric replicable nanogenerator with nanoscale surface-relief features (nano-PATERN) by using thermal nanoimprinting (Fig. 4c).^89^ The test results show that the nano-PATERN confers higher electrical output performance compared with the flat-PATERN.

Researchers also constructed micro/nanostructures on the surface of the tribo-layer by laser ablation. Compared with the template method, laser ablation yields greater accuracy and a shorter working period for creating the pattern on the surface. As shown in Fig. 4d, Huang *et al.* fabricated different micro/nano structures on the Cu and PDMS films by laser ablation technology.^90^ Laser scanning ablation technology is used to create micro/nano dual-scale structures in stripes and cones on Cu film surfaces. Additionally, micro-bowl structures in various sizes are fabricated on polydimethylsiloxane (PDMS) surfaces through single pulse irradiation. This unique surface topography effectively increases the contact area and achieves a 21-fold increase in power density compared to the TENGs without micro/nano-structures. Fig. 4e shows the fabrication procedure of a film-sponge-coupled TENG (FS-TENG) *via* direct ultraviolet laser ablation proposed by Cho *et al.*^91^ Various surface structures can be created on the surface of the PI film in one minute by ultraviolet laser ablation. Next, a sponge made from non-woven polyamide and silicone rubber is designed to have full contact with the micro-/nano-scale structures on the surface of the PI film. The FS-TENG demonstrates an open-circuit voltage of 48.19 V and a short-circuit current of 1.243 μA, reflecting a threefold improvement in electrical performance compared to the FS-TENG with a pristine PI film.

The methods mentioned above are generally used to make regular micro/nanostructures, while methods such as the electrostatic spinning process and others are used to build irregular micro/nanostructures on the surface of the tribo-layer. Sun *et al.* reported a fabricating nanofiber-based TENG (NF-TENG) (Fig. 4f).^92^ The NF-TENG consisted of the electrospun PA66/MWCNTs nanofibers film serving as the tribo-positive layer and the electrospun PVDF nanofibers film serving as the tribo-negative layer, respectively. Electrospun fibers offer the benefits of uniformity, high porosity, and a large surface area, which improves the output performance of TENGs. Xie *et al.* used the dynamic supercritical carbon dioxide (scCO_2_) foaming technique to fabricate TPU foams with micro-sized pores, as shown in Fig. 4g.^93^ The biomimetic wrinkles that are induced by the scCO_2_ flow field facilitate contact electrification and greatly enhance the triboelectric output performance.

The detailed output performance comparison of TENGs before and after the surface morphology modification is shown in Table 1. In summary, the surface morphological modification can efficiently enhance the output performance of TENGs. The modified TENG can collect energy from various mechanical movements more efficiently, making it suitable for a wider range of application scenarios. However, the method provides only minimal performance improvement and a limited impact on the electron withdrawing/donating ability of triboelectric materials. Therefore, in the following review, we will introduce several strategies to improve the electron withdrawing/donating abilities of triboelectric materials.

**Table 1:** The summary of TENG with surface morphological modification

Surface modification method	Process	Special structure	Before modification		After modification		Ref.
			*V*_OC_	*I*_SC_	*V*_OC_	*I*_SC_	
Surface pattering	Template method	Pyramid	3.4 V	0.16 μA (0.33 Hz)	10 V	0.7 μA (0.33 Hz)	18
		Nanopillar	295 V	10.4 μA (5 Hz)	568 V	25.6 μA (5 Hz)	94
	Thermal nanoimprint	Nano-PATERN	1.24 V	0.102 mA m^−2^	3.19 V	0.722 mA m^−2^	89
Laser ablation	Laser direct writing	PDMS micro-bowls	5.34 V	0.51 μA (1.5 Hz)	22.04 V	2.6 μA (1.5 Hz)	90
		Cu micro/nano-cones					
	Direct ultraviolet laser ablation	Square pattern	16.82 V	0.52 μA (5 Hz)	48.19 V	1.243 μA (5 Hz)	91
Other methods	Electrospinning	Nanofiber	—	—	142 V	15.5 μA (5 Hz)	92
	Supercritical carbon dioxide foaming	Porous polymer	—	—	78 V	0.5 μA (4 Hz)	93

### 4.2 Chemical modification

Chemical modification refers to introducing chemical functional groups on the surface of the triboelectric material by chemical reaction, which aims to change the electronic structure of the material and thereby increase the transferred charge during friction.

**Fig. 5:**
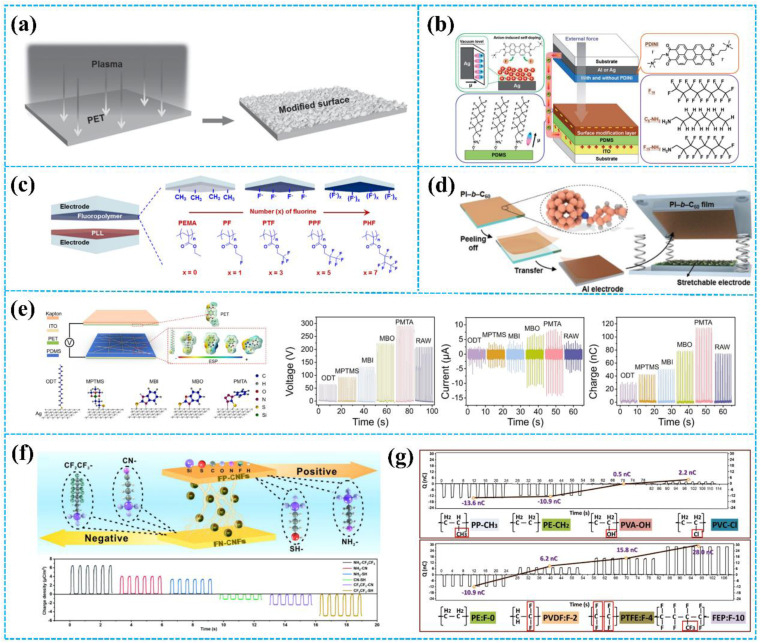
(a) Surface fluorination of the PET film by inductive-coupled plasma etching. Reprinted with permission from ref. 95. Copyright 2015, Wiley. (b) Using an electrostatically self-assembled molecule 1*H*,1*H*-perfluoro-octylamine (F_15_–NH_2_) as the surface modification for the PDMS dielectric layer. Reprinted with permission from ref. 41. Copyright 2021, Royal Society of Chemistry. (c) TENGs based on fluorinated polymers with different kinds of fluorine units. Reprinted with permission from ref. 96. Copyright 2018, Elsevier. (d) Introduction of C_60_ to the PI film. Reprinted with permission from ref. 97. Copyright 2021, Royal Society of Chemistry. (e) Decorating different chemical functional groups (CFGs) through the SAM treatment. Reprinted with permission from ref. 98. Copyright 2023, Elsevier. (f) Contact electrification performance of CNFs with chemically tailored molecular surface modification. Reprinted with permission from ref. 99. Copyright 2021, Elsevier. (g) Comparison of different functional groups on the contact electrification of polymers. Reprinted with permission from ref. 82. Copyright 2020, Wiley.

Importing appropriate chemical functional groups is the key to improving the output performance of TENGs. Fluorine has garnered significant attention from researchers due to its strong electron-absorbing ability. In Fig. 5a, Li *et al.* reported chemical modification of a PET film *via* inductive-coupled plasma etching.^95^ The modification uses a gas mixture of carbon tetrafluoride (CF_4_) and oxygen (O_2_) as the plasma source. The plasma etching not only resulted in surface fluorination, but also developed micro/nanostructures on the surface of the PET film. Therefore, the TENG based on the modified PET film achieved a maximum *V*_OC_ of ≈220 V, an *I*_SC_ of ≈45 μA, and an induced charge of ≈130 nC, which are much higher than the unmodified PET film. Except for plasma etching, electrostatic self-assembly was also used to introduce the chemical functional groups. Based on the electrostatic self-assembly, Yang *et al.* imported the molecule 1*H*,1*H*-perfluoro-octylamine (F_15_–NH_2_) as the chemical functional group onto the surface of the PDMS layer (Fig. 5b).^41^ Perfluoroalkyl chains have a tendency to accumulate at the air interface due to their high electronegativity, which improves the charge transfer efficiency between the electrode and tribo-layer. The *V*_OC_, *I*_SC_, and power density of the TENG based on the treated PDMS film are 1392 V, 158.4 μA, and 57.1 W m^−2^, respectively. The reports mentioned above have shown that surface fluorination is an efficient method to increase the output performance of TENGs. Moreover, the output performance of TENGs is also affected by the molecular structures of fluorinated polymers and the number of fluorine units. Kim *et al.* synthesized a TENG based on fluorinated polymers with different kinds of fluorine units (Fig. 5c).^96^ The results show that the dielectric constant and the triboelectric performance of the fluorinated polymers increase with increasing fluorine units.

In addition to the fluorinated group, the researchers introduced different kinds of chemical functional groups on the tribo-layer surface. As shown in Fig. 5d, Lee *et al.* fabricated a C_60_-containing block polyimide (PI-*b*-C_60_) by the cycloaddition reaction of azide-containing PI with C_60_.^97^ C_60_ is recognized as an effective electron acceptor molecule. Therefore, the PI-*b*-C_60_ film shows highly electronegative ability. The TENG based on PI-*b*-C_60_ achieved a superior charge density of over 300 μC m^−2^. Interestingly, based on its excellent triboelectric properties, the TENG based on PI-*b*-C_60_ also performs well in non-contact applications. Furthermore, Shin *et al.* introduced a series of halogens on the surface of the PET film.^100^ The PET surface was functionalized with halogen (Br, F, and Cl)-substituted phenyl or aminated molecules, resulting in a diverse range of triboelectric properties. Importantly, testing results show that the transferred charge density was arranged in the order of electron affinity. In 2019, Lee *et al.* prepared sulfur backbone-based inorganic polymers.^101^ Based on the high electron affinity of sulfur, the open-circuit voltage output of the TENG can reach 1366 V and light 630 LEDs under a minimal external force of ∼30 N. Moreover, Yao *et al.* introduced nitro groups and methyl groups on cellulose nanofibrils (CNF) to change the tribopolarities of CNF.^102^ Specifically, due to the nitro group having excellent electron-absorbing ability and the methyl group having excellent electron-releasing ability, the methyl-CNF and nitro-CNF have tribopositivity and tribonegativity, respectively. The TENG, based on the methyl-CNF as the positive tribo-layer and the nitro-CNF as the negative tribo-layer, demonstrated an average voltage output of 8 V and a current output of 9 μA.

To deeply explore the effect of chemical modification on the surface charge density, Lei *et al.* decorated different chemical functional groups (including octadecanethiol (ODT), 3-mercaptopropyl trimethoxysilane (MPTMS), 2-mercaptobenzimidazole (MBI), 2-mercaptobenzoxazole (MBO), and 1-phenyl-5-mercaptotetrazole (PMTA)) on AgNW-based transparent conductive films by self-assembled monolayer (Fig. 5e).^98^ According to the HOMO/LUMO theory, the low-lying LUMO of the acceptor will dominate the electron transport. The electron-absorbing ability follows the sequence: PMTA > MBO > MBI > MPTMS > ODT. Therefore, TENG based on the PMTA-modified PDMS layer achieved the best output performance, with the maximum *V*_OC_, *I*_SC_, and *Q*_SC_ reaching 290 V, 22.6 μA, and 114 nC, respectively. As shown in Fig. 5f, Liu *et al.* introduced different functionalities (electron-withdrawing and electron-donating groups) on the cellulose nanofibrils (CNFs).^99^ The charge density of CNFs is weakened by the introduction of electron-withdrawing groups and enhanced by the introduction of electron-donating groups. The strength of the electron-donating ability of each functional group is given by: –NH_2_ > –SH > –CN > –CF_2_CF_3_. Similarly, Li *et al.* ranked the electron-withdrawing ability of halogen groups (in Fig. 5g).^82^ It follows the order: CH_3_ < H < OH < Cl < F. This makes a great contribution to further elucidating the relationship between functional groups and the triboelectric effect.

The detailed output performance comparison of TENGs before and after chemical modification is shown in Table 2. Among the results, 1-phenyl-5-mercaptotetrazole is the most effective chemical to improve the output performance of TENGs. However, it is important to note that the choice of chemicals and their application methods should be tailored to the specific type of TENG and the materials used. Additionally, the environmental impact and long-term stability of the chemicals should also be considered when enhancing TENGs' output performance.

**Table 2:** The summary of TENG with chemical modification

Chemical modification process	Functional group	Before modification			After modification			Ref.
		*V*_OC_	*I*_SC_	*Q*_SC_	*V*_OC_	*I*_SC_	*Q*_SC_	
Inductive-coupled plasma etching	CF_*n*_	86.1 V	19.4 μA (1.66 Hz)	46.9 nC	217.2 V	46.3 μA (1.66 Hz)	124.4 nC	95
Electrostatically self-assembled	C_8_–NH_2_	182 V	19.6 μA (14 Hz)	10.8 nC	186 V	20.2 μA (14 Hz)	11.4 nC	41
	F_18_				287 V	21.2 μA (14 Hz)	14.6 nC	
	F_15_–NH_2_				540 V	44.8 μA (14 Hz)	47.9 nC	
Cycloaddition reaction	C_60_	158.8 V	32.1 mA m^−2^ (3 Hz)	32.7 μC m^−2^	505.1 V	117.9 mA m^−2^ (3 Hz)	107.2 μC m^−2^	97
Self-assembled monolayer	1-Phenyl-5-mercaptotetrazole	90 V	5 μA (1 Hz)	35 nC	290 V	22.6 μA (1 Hz)	114 nC	98
Chemically tailored molecular surface modification	NH_2_–	50.3 V	—	8.77 μC m^−2^	76 V	—	13.2 μC m^−2^	99
	SH–				65 V	—	10.75 μC m^−2^	
	CN–				42.2 V	—	7.9 μC m^−2^	
	CH_3_CH_2_–				33.1 V	—	6.5 μC m^−2^	

In summary, the output performance of TENGs is greatly enhanced by the introduction of suitable chemical functional groups. Furthermore, some chemical functional groups not only increase the surface charge density, but also improve the moisture resistance and temperature resistance of triboelectric materials, making the TENG maintain high output performance in harsh environments.^102–105^ Additionally, chemical modification broadens the range of available materials for high-performance TENGs.^95,106^

### 4.3 Nanocomposite

#### 4.3.1 Simple blending

The dielectric constant of the triboelectric material also plays an important role in the output performance of TENGs. Doping nanomaterials into the polymer is an effective method to improve the dielectric constant of the triboelectric material, thereby improving the TENG's output performance.^107–110^

**Fig. 6:**
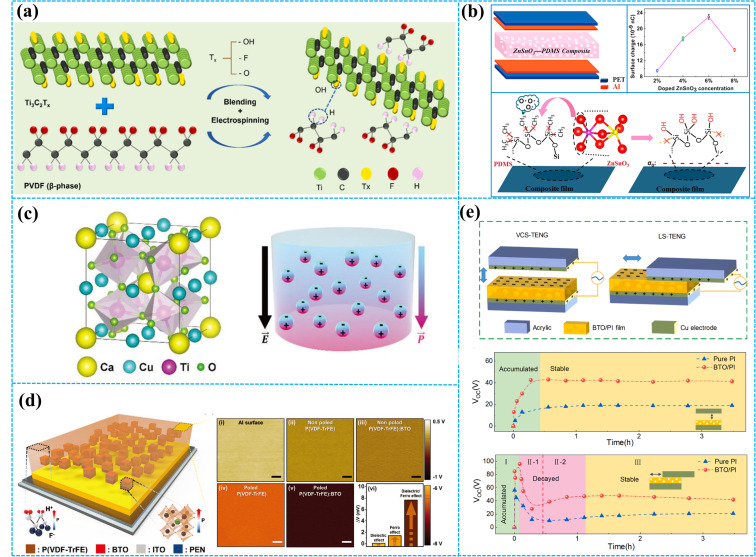
(a) Fabrication process of the PVDF/MXene composite film and property of PVDF with different MXene concentrations. Reprinted with permission from ref. 114. Copyright 2021, Elsevier. (b) Structure of ZnSnO_3_–PDMS-based TENG and schematic illustration of the charges in the molecular structure. Reprinted with permission from ref. 115. Copyright 2015, Elsevier. (c) Chemical structure of CCTO and the dielectric constant of the BMF–CCTO composite film under an electric field. Reprinted with permission from ref. 42. Copyright 2020, Wiley. (d) Schematic description of a ferroelectric composite-based TENG and KPFM study of the ferroelectric composite film. Reprinted with permission from ref. 116. Copyright 2017, Wiley. (e) The output performance of the BTO/PI nanocomposite film-based VCS-TENG and LS-TENG. Reprinted with permission from ref. 117. Copyright 2022, Elsevier.

By adding a small amount of 2D conductive nanomaterials (such as graphene and MXene) to the polymer matrix, the dielectric constant of the composite material will be significantly improved due to the conductive two-dimensional planar structure of the materials and the insulating polymer together constituting many microcapacitors, improving the ability of the composite material to store charge.^111–113^ As shown in Fig. 6a, Bhatta *et al.* doped MXene nanosheets into the PVDF matrix.^114^ The dielectric constant of the PVDF composite film increased as the MXene concentration increased. For the MXene concentration of 25 wt%, the dielectric constant of the PVDF composite film is 44.1, which is much higher than that of the pristine PVDF (13.35). Therefore, the demonstrated TENG based on the PVDF composite film can reach maximum *V*_OC_, *I*_SC_, and transferred charge values of 724 V, 163.6 μA, and 182 nC, respectively.

Except for conductive nanomaterials, high-dielectric materials are also appropriate for doping selection. Wang *et al.* fabricated lead-free ZnSnO_3_ nanocubes@polydimethylsiloxane (PDMS)-based TENG by dispersing ZnSnO_3_ nanocubes into PDMS (Fig. 6b).^115^ The TENG based on a composite PDMS film with a doped ZnSnO_3_ concentration of 6 wt% achieved the best output performance, with an output current that was up to 6.2 times greater than that of the pure PDMS film-based TENG. In Fig. 6c, Kim *et al.* added high permittivity CaCu_3_Ti_4_O_12_ (CCTO) particles into butylated melamine formaldehyde (BMF).^42^ CCTO particles with a high permittivity of 7500 can induce a strong internal polarization within the dielectric material when subjected to an electric field generated by triboelectric charges. Under identical electric field conditions, the BMF–CCTO 1 wt% composite exhibited three times the internal polarization of pure BMF. A rotation-type freestanding mode TENG based on the BMF–CCTO 1 wt% composite film generated a high RMS voltage and current density of 268 V and 25.8 mA m^−2^, respectively.

In recent years, ferroelectric materials have attracted much attention because of their high dielectric constant and ferroelectric effect. Suo *et al.* prepared a novel hybrid piezo/triboelectric nanogenerator based on the BaTiO_3_ (BTO)/PDMS composite film.^118^ The PDMS composite film with a BTO concentration of 20 wt% showed the best performance due to its ferroelectric polarization strength and dielectric constant. This work confirmed that piezoelectric and triboelectric effects can coexist in a single material component and interact to improve the electric output performance. As shown in Fig. 6d, Seung *et al.* reported a nanocomposite material system that consists of a high-dielectric ceramic material, barium titanate (BTO), and a ferroelectric copolymer matrix, poly(vinylidenefluoride-*co*-trifluoroethylene) (P(VDF–TrFE)).^116^ The surface charge potential dramatically increased due to the electrically induced ferroelectric polarization inside the P(VDF–TrFE) with dielectric BTO NPs. Under the combined action of triboelectric and ferroelectric effects, the TENG based on BTO/PVDF–TrFE composite films achieved a boosted power-generating performance that improved by about 150 times compared with typical triboelectric material-based devices. Li *et al.* selected BaTiO_3_ nanoparticles (BTO NPs) as the doping material, which was dispersed into the PI matrix, as displayed in Fig. 6e.^117^ Interestingly, the ideal doping mass concentration of BTO NPs for maximizing the electrical performance depends on the operating mode of TENGs. For LS-TENG, 5 wt% BTO NPs show the best performance, doubling the steady-state open-circuit voltage compared to that of the pure PI film. Similarly, 18 wt% BTO NPs display the best results for CS-TENG with the steady-state open-circuit voltage doubled compared with the pure PI film.

#### 4.3.2 Multilayer structure of the composite film

It is a common strategy to prepare polymer-based composite films by incorporating high-dielectric nanomaterials for high-performance tribo-layers.^119–121^ However, it is a challenge to uniformly disperse high-dielectric nanomaterials in polymers.^122^ A valid way to achieve a high dielectric constant and output performance is by fabricating high-dielectric nanomaterials/polymer composites with multilayer structures. Firstly, interfacial polarization can form at the interfaces between different layers, resulting in charge accumulation at the multilayer interfaces and enhancing the dielectric constant. Secondly, polymer film on the high dielectric constant layer surface can effectively inhibit breakdown and charge leakage. Last but not least, the multilayer structure prevents high dielectric material agglomeration and minimizes defect formation.

**Fig. 7:**
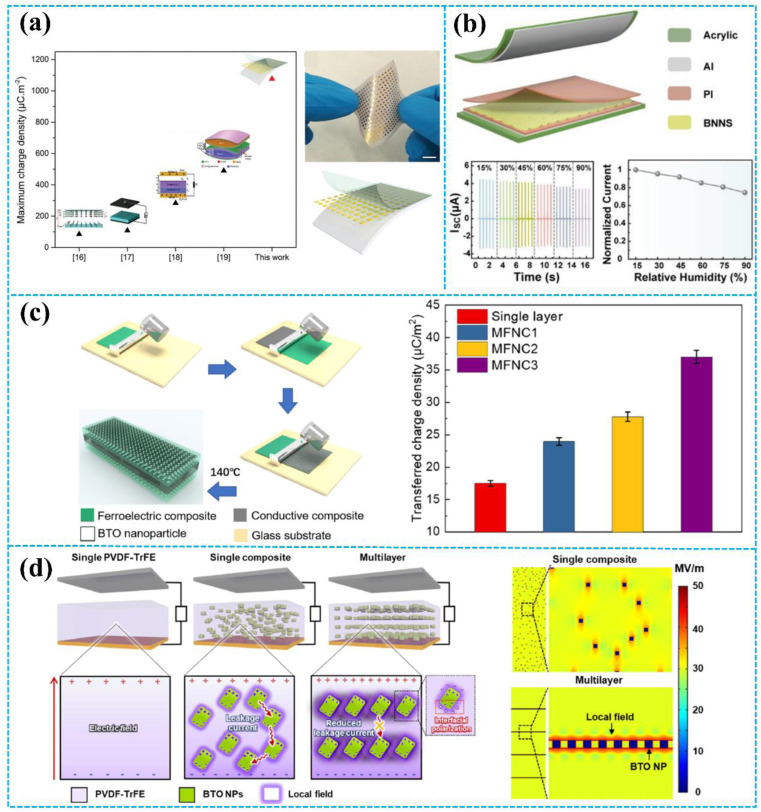
(a) Design, structure, and working mechanism of the insulator–metal–insulator TENG. Reprinted with permission from ref. 123. Copyright 2020, Wiley. (b) A sandwich-shaped nanocomposite film-based TENG and the output performance of the TENG. Reprinted with permission from ref. 124. Copyright 2022, American Chemical Society. (c) The fabrication process and output performance of MFNCs. Reprinted with permission from ref. 49. Copyright 2022, Elsevier. (d) Schematic of the three different types of composite film and the FEM simulations of the electric field distribution in the single and multilayered PVDF–TrFE/BTO composite films. Reprinted with permission from ref. 125. Copyright 2020, American Chemical Society.

Ravichandran *et al.* developed a multilayer flexible composite structure by employing an insulator–metal–insulator architecture in place of a single insulator material (in Fig. 7a).^123^ The multilayer composite structure consists of an intermediate gold (Au) metal inclusion sandwiched in-between a charge generation and retention layer, parylene-C (PaC), and polytetrafluoroethylene (PTFE). The metal inclusion acts as a charge storage site and its storage is several orders of magnitude greater than that of the bare insulator, enabling it to accumulate the triboelectric charge generated at each cycle. Based on the unique structural design, the TENG exhibited great output performance, resulting in a maximum charge density of 1076.56 μC m^−2^ and a maximum output power density of 4.8 W m^−2^. In Fig. 7b, Pang *et al.* fabricated a TENG based on a sandwich-structured polyimide (PI)/boron nitride nanosheet (BNNS)/PI nanocomposite film (PBP).^124^ The introduction of the BNNS interlayer significantly enhances the triboelectric performance of the PI nanocomposite film. Aluminum (Al) and PBP multilayers were used as the positive and negative contacting triboelectric layers, respectively. The short-circuit current of the TENG based on the PBP multilayer was 4.5 μA, which was 5 times that of the TENG without the BNNS interlayer. In addition, at an external load resistance of 10 MΩ, the TENG based on the PBP multilayer achieved a maximum power density of 21.4 μW cm^−2^.

Similarly, to increase the TENG's output power, Chai *et al.* prepared multilayered ferroelectric nanocomposites (MFNCs) as negative tribo-layer, as shown in Fig. 7c.^49^ A conductive interlayer (multiwalled carbon nanotube (MWCNT) filled P(VDF–TrFE)) was sandwiched in two ferroelectric nanocomposites (BaTiO_3_/P(VDF–TrFE)). The output performance of the TENG was greatly improved, resulting in a maximum transferred charge density and power density of 105.70 μC m^−2^ and 7.21 W m^−2^ respectively, at 2 Hz, due to the enhanced polarization of ferroelectric materials by introducing a conductive interlayer. Moreover, the conductive interlayer can also trap triboelectric surface charges. Additionally, Cao *et al.* prepared X–Y–X sandwich structured copper calcium titanate/polyimide (CCTO/PI) nanocomposites for TENG by using a layer-by-layer casting method.^126^ In the X–Y–X sandwich-structure, polyimide (PI) with a high dielectric constant copper calcium titanate (CCTO) and pure PI film were used as the outer layer and charge storage layer, respectively. By introducing a charge-storage layer of PI, the output performance was also improved. The *V*_OC_, *I*_SC_, and *Q*_SC_ values of the TENG based on the X–Y–X sandwich-structure were 96.6 V, 5.6 μA, and 30.8 nC, respectively.

Ferroelectric composites have been found to improve the performance of TENGs, but their output performance is hindered by randomly dispersed particles. Therefore, Park *et al.* introduced a high-performance TENG based on ferroelectric multilayer nanocomposites with alternating poly(vinylidenefluoride-*co*-trifluoroethylene) (PVDF–TrFE) and BaTiO_3_ (BTO) nanoparticles (NP) layers, as shown in Fig. 7d.^125^ From the COMSOL Multiphysics result, it is clear that the multilayer structure featuring BTO NPs on the coplanar layer facilitates a more efficient connectivity of interfacial charges at closer distances compared to composites with randomly dispersed BTO NPs, which leads to a significantly enhanced local field while boosting the ferroelectric polarization of the polymer. Moreover, with characteristics that induce stress concentration, the dielectric constant of multilayers consisting of alternating soft/hard layers surpasses that of single PVDF–TrFE/BTO nanocomposites (15.9) and pure PVDF–TrFE films (13.9). Consequently, the multilayered TENG showed 2.3 and 1.5 times higher current densities than pure PVDF–TrFE and PVDF–TrFE/BTO nanocomposites without a multilayer structure, respectively.

The detailed output performance comparison between TENGs based on a pristine polymer and TENGs based on a composite film is shown in Table 3. Among the results, the TENG based on ferroelectric materials/polymer composite films shows the best output performance by coupling the triboelectric effect and the piezoelectric effect. Additionally, the TENG based on the multilayer structure has better output performance compared with the TENG based on single blending (single layer). Thus, the surface charge density of the triboelectric materials is effectively enhanced by doping nanomaterials into the polymer. By designing a multilayer composite membrane structure, the dielectric properties of the single-layer composite film are efficiently improved. Furthermore, the coupling of the triboelectric effect and piezoelectric effect is achieved by doping ferroelectric nanoparticles, thereby efficiently increasing the surface charge density and hence boosting TENGs' output performance.

**Table 3:** The summary of TENG with nanocomposite film^a^

Nanocomposite	Composite film	Inclusions	Pristine polymer				Composite film				Ref.
			*V*_OC_	*I*_SC_	*Q*_SC_	Output power	*V*_OC_	*I*_SC_	*Q*_SC_	Output power	
Simple blending	Ag/chitosan	Ag nanowires	17.1 V	0.9 μA (3 Hz)	7.2 nC	—	47.9 V	4.1 μA (3 Hz)	17.5 nC	137.6 mW m^−2^	127
	MWCNTs/chitosan	MWCNTs	14.4 V	0.3 μA (2 Hz)	5.8 nC	—	85.8 V	8.7 μA (2 Hz)	29 nC	180 mW m^−2^	128
	LM/PDMS	Liquid metal (LM)	52 V	3.9 μA (3 Hz)	30 nC	36 mW m^−2^	210 V	10.3 μA (3 Hz)	119 nC	1020 mW m^−2^	129
	MXene/PVDF	MXene	470 V	89.4 μA (8 Hz)	101 nC	—	724 V	163.6 μA (8 Hz)	182 nC	11.213 W m^−2^	114
	MoS_2_/nylon	MoS_2_	120 V	130 μA m^−2^ (0.8 Hz)	42 μC m^−2^	6 mW m^−2^	270 V	645 μA cm^−2^ (0.8 Hz)	20.2 nC cm^−2^	50 mW m^−2^	130
	MoS_2_/PVDF										
	BNNs/PDMS	BNNs	935 V	63.9 mA m^−2^ (5 Hz)	86 μC m^−2^	33.6 W m^−2^	1870 V	230 mA m^−2^ (5 Hz)	234 μC m^−2^	100 W m^−2^	131
	MOFs/PDMS	Zr-based MOFs	52.8 V	4.1 μA (1 Hz)	7.5 nC	—	130 V	7.1 μA (1 Hz)	18.1 nC	—	132
	ZnSnO_3_/PDMS	ZnSnO_3_	224 V	3.2 μA (2 Hz)	3.8 nC	—	330 V	16 μA (2 Hz)	40.5 nC	3 mW	115
	BaTiO_3_/P(VDF–TrFE)	BaTiO_3_	93 V	40 μA (0.5 Hz)	—	—	331 V	300 μA (0.5 Hz)	—	6.4 mW	116
Multilayer structure	PI-MoS_2_:PI-PI (sandwich structure)	MoS_2_:PI layer	30 V	—	50 nC	0.21 W m^−2^	400 V	—	200 nC	25.7 W m^−2^	107
	IZO–PDMS (bilayer structure)	IZO layer	35 V	20 μA cm^−2^ (1 Hz)	28 μC m^−2^	0.75 mW cm^−2^	140 V	180 μA cm^−2^ (1 Hz)	108 μC m^−2^	25 mW cm^−2^	133
	PI–BNNs–PI (sandwich structure)	BNNs layer	9.3 V	0.9 μA (2 Hz)	2.8 nC	14.3 mW m^−2^	65.9 V	4.5 mA (2 Hz)	17.2 nC	214 mW m^−2^	124
	BTO/P(VDF–TrFE)–MWCNTs/P(VDF–TrFE)–BTO/P(VDF–TrFE) (sandwich structure)	MWCNTs/P(VDF–TrFE) layer	70 V	4.6 μA	33.4 μC m^−2^	0.11 W m^−2^	131.2 V	10.4 μA	65.4 μC m^−2^	7.21 W m^−2^	49

a MWCNTs: multi-walled carbon nanotubes; BNNs: boron nitride nanosheets; IZO: indium zinc oxide.

### 4.4 Charge injection

The output performance of TENGs is strongly influenced by their surface charge density. Injecting charge into the surface of the tribo-layer is the most direct and effective way to increase the surface charge density.

**Fig. 8:**
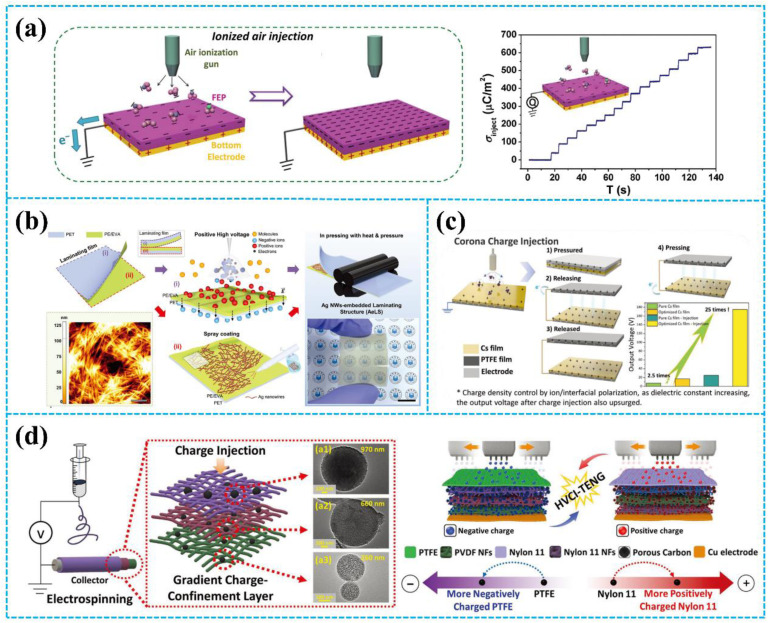
(a) Injecting negative ions onto the FEP surface from an air-ionization gun and the output performance of the treated FEP film. Reprinted with permission from ref. 134. Copyright 2014, Wiley. (b) Fabrication process of AeLS with i-CDT. Reprinted with permission from ref. 43. Copyright 2023, Wiley. (c) Schematic diagram of the corona charge injection and structure/working principle of the TENG. Reprinted with permission from ref. 135. Copyright 2022, Wiley. (d) Schematic of the fabrication process of the TENG and injection of the negative and positive charges to PTFE and nylon-11, respectively. Reprinted with permission from ref. 136. Copyright 2022, Wiley.

Wang *et al.* utilized an air-ionization gun to bring the negative charges onto the surface of a FEP film (Fig. 8a).^134^ The air-ionization gun could generate positive and negative charges by ionizing air inside the gun. To monitor the negative charges on the FEP surface, researchers used a Coulomb meter to measure the charge flow from the ground to the bottom electrode. The result shows that each ion injection event transfers charges with a charge density of approximately 40 μC m^−2^ from the ground to the bottom electrode, introducing charges of the same density onto the FEP surface. After the 17 consecutive instances of charge injection, the negative static charge density on the FEP surface finally reached ∼630 μC m^−2^. In this study, the maximum surface charge density for the CS-mode TENGs can be determined by comparing the threshold voltage for the air breakdown with the actual voltage drop (*V*_gap_) across the air gap. The *V*_gap_ has the following relationship with the gap distance (*x*):^134^7
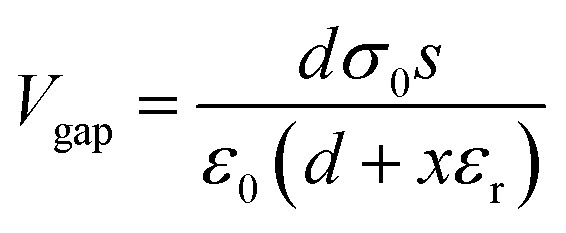
where *d* is the thickness of the FEP film, *ε*_r_ is the relative permittivity of the FEP layer, *σ*_0_ is the surface charge density, and *ε*_0_ is the vacuum permittivity. The air breakdown voltage (*V*_AB_) curve can be described according to the empirical formula of Paschen law:^137–139^8
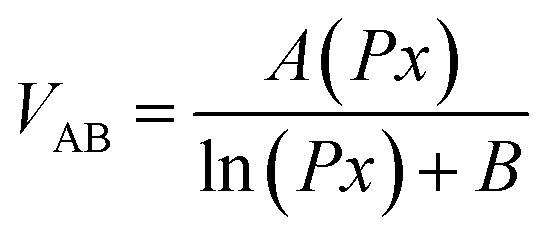
where *P* is the atmospheric pressure, *x* is the gap distance, and *A* and *B* are the constants determined by humidity, temperature, *etc.* in the environment. During the contact-separation process, to obtain the maximum surface charge density, the *V*_gap_ must be smaller than the *V*_AB_. Therefore, in the whole contact-separation process, the relationship between *V*_gap_ and *V*_AB_ should be satisfied for:^134^9
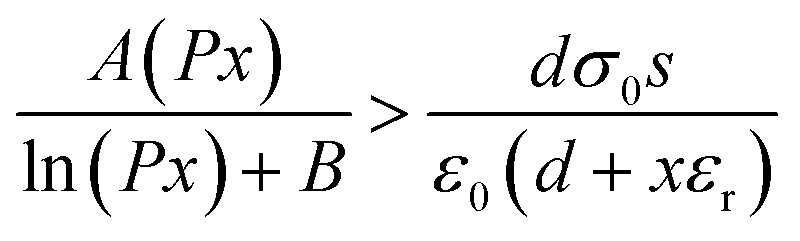


According to eqn (8), the maximum surface charge density (*σ*_max_) for the CS-mode TENGs is:10
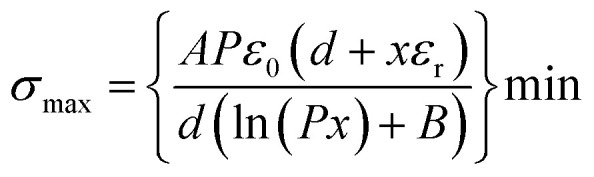


From this equation, it is proposed that thinner dielectric films are the preferred choice for achieving a higher surface charge density. Liu *et al.* introduced the surface charges to the surface of different polymer films using an air-ionization gun to investigate the surface charge decay trend of the TENGs in a high-humidity environment.^140^ It was discovered that increasing the hydrophobicity of dielectric materials can lead to higher surface charges, particularly in high humidity conditions. The TENG based on the PTFE film retained 90% of its initial output performance after 5000 cycles under 90% relative humidity.

In addition to the air-ionization gun, a corona discharge treatment (CDT) can be used to deposit charges onto the surface of the tribo-layer. As shown in Fig. 8b, Park *et al.* reported an Ag nanowire (NW)-embedded laminating structure (AeLS) for ionic charge injection by CDT.^43^ Based on their unique laminated structure, Ag nanowires dissipate positive charges, allowing negative ions to accumulate on the outermost surface. The testing results show that the AeLS with CDT exhibited higher durability and resistance to moisture and water molecules. The output current, charge, and power density of the TENG based on AeLS were ≈146 μA, ≈300 nC, and ≈1.6 W m^−2^, respectively. In addition to introducing charges onto the surface of the tribo-layer, the corona discharge treatment can optimize the polarization of triboelectric materials. Sun *et al.* enhanced the interfacial polarization of the chitosan blends by corona charge injection to enhance the output performance (Fig. 8c).^135^ During the corona charge injection, the ion in the chitosan blends will move in the direction of the electric field, which leads to enhanced polarization and then increases the dielectric constant of the chitosan blends. Therefore, after corona charge injection, with the increasing dielectric constant and surface charge density, the TENG based on the treated chitosan blends exhibited an output voltage that is 25 times (200 V) higher than that of the TENG based on the initial film.

In another study, Cha *et al.* proposed the improvement of the output performance of TENGs through introducing charge to the gradient charge-confinement layer based on electrospinning fibers by high-voltage charge injection (HVCI), as displayed in Fig. 8d.^136^ Each successive nanofibrous layer in the gradient charge-confinement layer included a larger number of mesoporous carbon spheres (mCSs) in increasing size. The gradient distribution of mCSs facilitated the movement of injected charge from the surface to the inner layers of the nanofibrous structure. When an external field of 7 kV was applied upon charge injection, the surface charge density on the gradient-charge confinement layer increased approximately 7.5 times compared to the case without mCSs. Consequently, the output voltage of the TENG was 600 V after charge injection, which represented an increase of ≈40 times compared to the output before charge injection.

However, the traditional charge injection process is more complex compared to the simple contact electrification process, and may require additional equipment support, such as an air-ionization gun, a high-voltage source for corona charge injection, and others. Therefore, developing a new simple charge injection method is urgent.

**Fig. 9:**
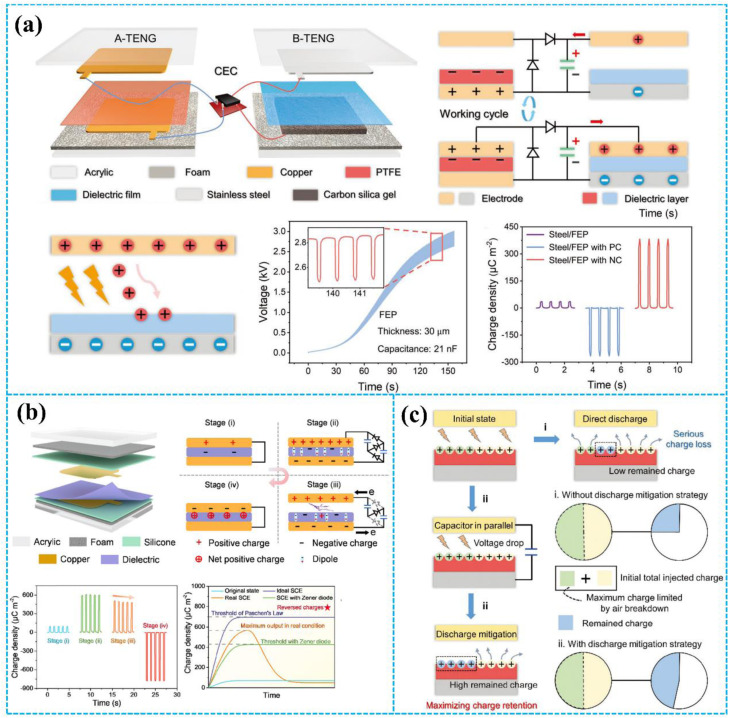
(a) Structure and principle of charge injection enabled by air breakdown achieved by a half-wave charge excitation circuit. Reprinted with permission from ref. 50. Copyright 2022, Wiley. (b) Theory analysis for the charge reversion process. Reprinted with permission from ref. 37. Copyright 2023, Royal Society of Chemistry. (c) Discharge mitigation strategy to decrease the dissipation of the injected surface charge. Reprinted with permission from ref. 141. Copyright 2023, Wiley.

In recent years, Wu *et al.* reported a simple and effective surface charge injection technology through a half-wave charge excitation circuit (CEC) (Fig. 9a).^50^ In previous work, it has been confirmed that the CEC can be used to improve the surface charge density of TENGs. In this research, the half-wave charge excitation circuit was used to provide a stable, high excitation voltage to achieve air breakdown and thus charge injection. A smaller capacitance in the CEC is beneficial for achieving a higher excitation voltage to improve the charge injection efficiency. Interestingly, the injected charge polarity on the surface of the dielectric polymers can be controlled by adjusting the connection mode of the CEC. After parameter optimization, the TENG based on the PI film reached an ultrahigh output charge density of 880 μC m^−2^ through this technology. Moreover, they also established a charge transfer model to quantify the surface charge density. This study enhances the understanding of the output charge density of TENGs, which is essential for enhancing the TENG performance.

Additionally, Guo *et al.* designed a high-performance TENG based on a charge reversion process generated by the electrostatic breakdown effect (Fig. 9b).^37^ This report used the high voltage generated by the voltage-multiplier circuit (VMC) to cause an electrostatic breakdown effect. The charge-reversion process could be divided into four stages. In the initial stage, the tribo-layer (PTFE film) carries a small amount of negative charge. In the second stage, the TENG starts charging the capacitors in the VMC, and the gap voltage of the TENG also starts to increase. In the third stage, with the increasing gap voltage of the TENG, the electric field between the upper electrode and the dielectric film of the TENG eventually reaches the threshold for air breakdown to occur, which leads to positive charge being transferred to the surface of the dielectric film. Therefore, in the fourth stage, when the VMC is removed, the surface charges of the dielectric film are reversed to a positive polarity. Based on the charge reversion process, the surface charge density of the PTFE-based TENG increased from 97 μC m^−2^ to 780 μC m^−2^, which surpassed the theoretical limit from Paschen's law.

The dissipation of the injected surface charge is still a limitation for charge injection. To improve the stability of the surface charge introduced by excessive charge self-injection, a step-by-step discharge mitigation strategy was proposed by Zhao *et al.*, as shown in Fig. 9c.^141^ They induced air ionization for charge injection onto the surface of the dielectric material by utilizing the directional high electric field generated by the charge excitation strategy of the voltage-multiplying circuit. At the operating frequency of 1.5 Hz, the injected charge on the dielectric film could be saturated in 22 seconds by using the charge excitation strategy. As shown in Fig. 9c(i), when the gap voltage of CS-TENG is higher than the air breakdown voltage, the discharge phenomenon occurs between the dielectric material and the electrode, leading to charge loss. Therefore, to reduce charge loss, a paralleling external capacitor was used to decrease the gap voltage of CS-TENG and then retain the maximum charge on the dielectric material surface, as displayed in Fig. 9c(ii). Based on the step-by-step discharge mitigation strategy, after charge injection, the TENG based on 7 μm thick PI film obtained an ultrahigh charge density of 1480 μC m^−2^ under the condition of 5% relative humidity.

To better demonstrate the current situation of charge injection, we compared the output performance of the TENGs before and after charge injection in Table 4. As can be seen from the output performance of TENGs in Table 4, these advanced methods of charge injection significantly improve the output performance of TENGs compared to TENGs without charge injection. According to the latest research, the charge self-injection strategy (charge injection technology through a half-wave charge excitation circuit) is the most effective way to improve the output performance of TENGs. Compared with the traditional charge injection method, it can achieve a higher surface charge density. With all that said, regardless of the used material, charge injection can increase the surface charge density of the triboelectric materials by directly increasing the number of charges. However, the injected charge is easily dissipated, which severely limits the application of charge injection in TENGs.

**Table 4:** The summary of TENG with charge injection

Charge injection method	Material	Before injection				After injection				Ref.
		*V*_OC_	*I*_SC_	*Q*_SC_	Output power	*V*_OC_	*I*_SC_	*Q*_SC_	Output power	
Ionized-air injection	FEP	200 V	18 mA m^−2^	50 μC m^−2^	—	1000 V	78 mA m^−2^	240 μC m^−2^	315 W m^−2^	134
High-voltage charge injection	Nylon 11	15.2 V	1.84 μA	—	—	600 V	12.8 μA	—	5.83 mW	136
	PTFE									
Ion-injection	PTFE/PEO	77 V	1.6 mA m^−2^ (5 Hz)	13.5 μC m^−2^	—	900 V	20 mA m^−2^ (5 Hz)	149 μC m^−2^	9 W m^−2^	142
Tunneling electron injection	FEP	—	—	120 μC m^−2^	0.49 W m^−2^	—	—	252 μC m^−2^	2.08 W m^−2^	143
Prior-charge injection	PVDF	584 V	15.3 mA m^−2^	79 μC m^−2^	—	1008 V	32.1 mA m^−2^	121 μC m^−2^	—	144
Charge injection enabled by air breakdown	PI	—	—	21 μC m^−2^	0.09 W m^−2^	—	40 mA m^−2^ (1 Hz)	880 μC m^−2^	9.04 W m^−2^	50
Charge reversion process	PTFE	—	4 mA m^−2^ (1 Hz)	97 μC m^−2^	1.5 mW m^−2^	—	81.7 mA m^−2^ (1 Hz)	720 μC m^−2^	89.4 mW m^−2^	37
Charge self-injection strategy	PI	—	2.8 mA m^−2^ (3 Hz)	23 μC m^−2^	86.9 mW m^−2^	960 V	350 mA m^−2^ (3 Hz)	1480 μC m^−2^	86 W m^−2^	141

## 5 Materials design for controlling charge loss

Except for charge generation, charge loss is also an essential factor that determines the output performance of TENGs.^139,145^ Charge loss can be divided into two parts: air breakdown and charge drift in the triboelectric materials.^39,83^ In this section, we will introduce two strategies to improve the output performance of TENGs: inhibition of air breakdown and utilization of charge drift.

### 5.1 Inhibition of air breakdown

In the actual TENGs output test, air breakdown is widespread and greatly affects the output performance of TENGs. Wang *et al.* utilized a dual dielectric layer to inhibit air breakdown.^139^ PVDF is chosen for the air breakdown suppression layer due to its high relative permittivity, while PI is chosen for the dielectric charge leakage suppression layer because of its low dielectric charge leakage. By simultaneously suppressing air breakdown and dielectric charge leakage, the maximum peak power density at 2 Hz is 61.3 W m^−2^ and the output charge density of the TENG is 2.2 mC m^−2^. To suppress air breakdown under high charge density conditions, Liu *et al.* demonstrated a new triboelectric polymer, poly(vinylidene fluoride–trifluoroethylene–chlorofluoroethylene) (P(VDF–TrFE–CFE)).^146^ The high dielectric permittivity of P(VDF–TrFE–CFE) can inhibit air breakdown. The increased upper limit of air breakdown in TENGs leads to unprecedented levels of charge density and energy density. The charge density and energy density of the TENG can reach 8.6 mC m^−2^ and 0.808 J m^−2^ per cycle, respectively. These studies effectively suppressed air breakdown in the TENG, preventing triboelectric charge decay and boosting the TENG output.

Recent studies indicate that air breakdown is inevitable when the TENGs work in the air and limits the maximum surface charge density of the TENG.^147,148^ In addition to the working environment, the working mode of TENGs has an influence on air breakdown.^84,149^ Especially in freestanding triboelectric-layer (FT) mode, air breakdown greatly limits the output performance of the TENG.

**Fig. 10:**
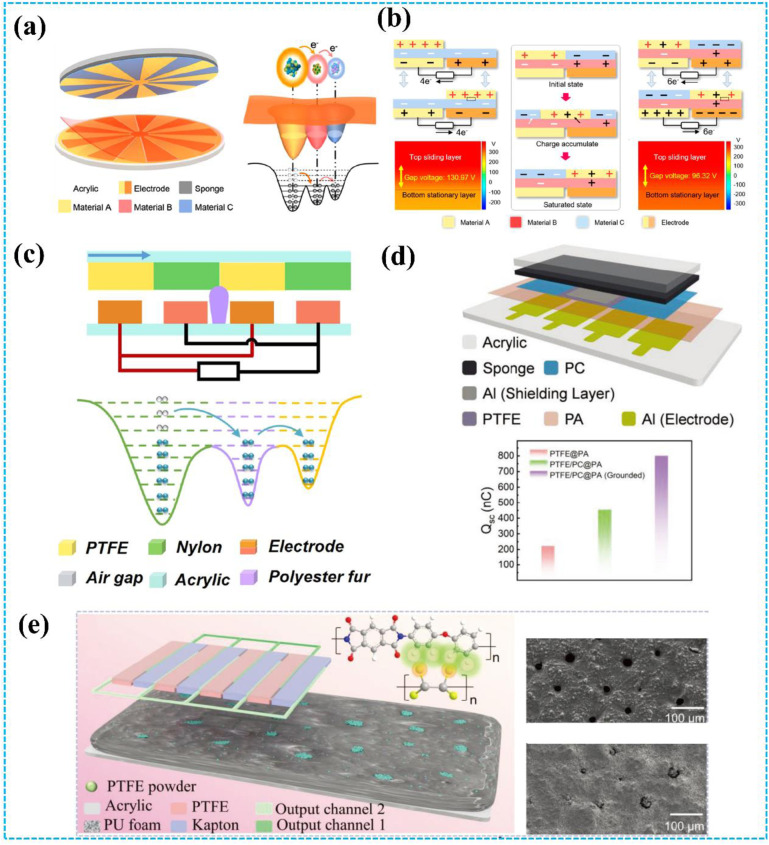
(a) Structural design of the TENG system and the electron-cloud-potential-well model during contact. (b) Schematics of the operating principle of the TENG system. Reprinted with permission from ref. 44. Copyright 2020, American Chemical Society. (c) The cross-section schematic of the TENG and the electron cloud potential well model for surface charge transfer. Reprinted with permission from ref. 45. Copyright 2021, Elsevier. (d) Structural schematic and output performance of TENG. Reprinted with permission from ref. 150. Copyright 2023, Wiley. (e) Structure of the ternary dielectric electrification TENG and SEM images of PU with/without PTFE powder. Reprinted with permission from ref. 151. Copyright 2024, Wiley.

In a recent study, Deng *et al.* reported a ternary electrification layered architecture TENG (TEL-TENG) system to inhibit air breakdown, as shown in Fig. 10a.^44^ Compared with previously reported binary electrification layered TENG (BEL-TENG), the upper rotator of the TEL-TENG is constructed by using copper and fluorinated ethylene propylene (FEP) as the two kinds of triboelectric materials and another triboelectric material Kapton was used for the stator of the TEL-TENG. Based on the unique structure design, the output performance of the TEL-TENG has been greatly improved, which leads to a 2.5-fold enhancement of the peak power compared to a BEL-TENG consisting of copper and FEP. Moreover, as the simulation results illustrate in Fig. 10b, a ternary electrification layered architecture can effectively reduce the electric field and then inhibit air breakdown. Additionally, in 2021, Li *et al.* proposed a polyester fur-reinforced rotary triboelectric nanogenerator (PFR-TENG), as displayed in Fig. 10c.^45^ This report also used the ternary electrification layered architecture, and polyester fur was chosen as the third triboelectric material. The PFR-TENG electric output remained at 100% after 100k cycles of continuous testing using partial soft-contact and non-contact modes, effectively reducing abrasion on the dielectric layers' surface and improving the stability of the TENG.

The ternary electrification layer (TEL) structure has been proven to be helpful in improving the output performance of TENG.^44,45^ However, air breakdown could also occur on the interface of the tribo-layers, which limits the maximization of the output of the TEL-TENGs. Therefore, to further improve the TENG's output, in 2023, An *et al.* reported a new design of TEL-TENG with a shielding layer and shrouded-tribo-area (SS-TEL-TENG) to prevent air breakdown (Fig. 10d).^150^ Air breakdown is effectively suppressed based on the grounded conductive shielding layer and the increased shrouded area. Through structure and material optimization, SS-TEL-TENG demonstrates a 3.59-fold increase in output charge compared to traditional S-TENG and a 1.76-fold increase compared to TEL-TENG. Additionally, the TEL structure can be used to improve the output performance of DC-TENG. As shown in Fig. 10e, Shan *et al.* proposed a multiple unit ternary electrification strategy based on triboelectrification and corona discharge.^151^ The triboelectric properties of the PU film can be controlled by adding PTFE powder, resulting in the realization of its electropositivity/electronegativity during friction with PTFE/Kapton. The output charge density of the optimized rotary TENG can reach 5.5 mC m^−2^ with an 82 μC transfer charge per round, which exceeds all previous sliding DC-TENG records.

To illustrate the present development of inhibition of air breakdown in TENGs, we summarized the output performance of TENGs using different methods to inhibit air breakdown in Table 5. From Table 5, it is clear that high-dielectric polymers can achieve high output performance by inhibiting air breakdown. Additionally, TENGs based on the FT working mode with a ternary electrification layered architecture have realized high output performance by inhibiting air breakdown. Inhibition of air breakdown has been achieved based on the triboelectric effect between three types of triboelectric materials, providing a new direction to reducing charge loss through material design.

**Table 5:** The summary of TENG with inhibition of the air breakdown

Strategy	Triboelectric material	Output performance				Ref.
		*V*_OC_	*I*_SC_	*Q*_SC_	Output power	
Dual dielectric layer	PVDF/PI (with charge excitation technology)	—	—	2.2 mC m^−2^	30.7 W m^−2^ Hz^−1^	139
High dielectric layer	P(VDF–TrFE–CFE) (with charge excitation technology)	—	—	8.6 mC/m^2^	0.77 W m^−2^ Hz^−1^	146
Ternary electrification layered architecture	Cu/FEP@Kapton (Kapton as the intermediate material)	993.6 V	21.7 μA	—	4.4 mW	44
	Nylon/PTFE@polyester fur (polyester fur as the intermediate material)	10 kV	15 μA	580 nC	201.83 mW	45
	PC/PTFE@PA (PA as the intermediate material)	—	25.8 μA	3.69 μC	25.4 mW	150

### 5.2 Utilization of charge drift

In previous studies, the leakage current of the tribo-layer has been a key factor limiting the output performance of TENGs. However, in recent research progress, researchers have started to use the leakage current of the tribo-layer to achieve charge migration and then improve the output of the TENG.

In 2018, Lai *et al.* investigated the transport and storage process of triboelectric charges in the tribo-layer by embedding criss-crossed gold layers in the near-surface of the tribo-layer.^121^ The main dynamic motion for triboelectric charges in the tribo-layer is the drift process caused by the electric field, and the direction of this process is vertically downward (from the surface to the inside of the material). The drift process of triboelectric charges decreases the surface charge density of the tribo-layer, which contributes to the further accumulation of triboelectric charges on the surface. The charge density of the TENG reaches 168 μC m^−2^, which is nearly 4 times the value of the TENG based on the pure polymer.

**Fig. 11:**
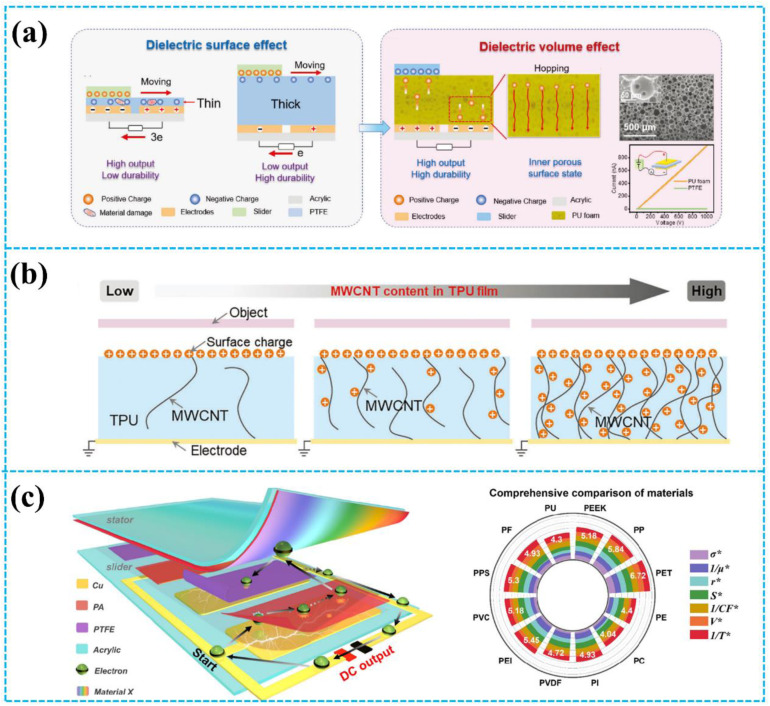
(a) Dielectric volume effect-based TENG. Reprinted with permission from ref. 46. Copyright 2023, Wiley. (b) Schematic diagrams and charge dispersion of CN-TENGs with different MWCNT contents. Reprinted with permission from ref. 47. Copyright 2024, Wiley. (c) Conceptual diagram of the TENG and comprehensive selection rules of intermediate triboelectric materials. Reprinted with permission from ref. 48. Copyright 2023, Royal Society of Chemistry.

Fu *et al.* reported a high output performance and durability of TENG by changing the dielectric surface effect into a volume effect through the leakage current of the millimeter-thick porous polyurethane (PU) foam film (Fig. 11a).^46^ The high leakage property of the porous film allows it to transfer electrical charges from the surface to the inside of the material, thus realizing high charge migration. Utilizing the strong charge migration characteristics of the porous PU foam film, the average power density of the TENG increased to 20.7 W m^−2^ Hz^−1^. Moreover, after 200 000 cycles, the output performance of the rotary-mode TENG did not change significantly, which showed high durability. This study offers a novel method to enhance TENGs' output performance and broaden the selection of materials for high-performance TENGs. Similarly, as shown in Fig. 11b, Sun *et al.* introduced multi-walled carbon nanotubes (MWCNT) in polyurethane (TPU) to realize charge migration.^47^ The charges generated by contact electrification could be transferred from the surface of the TPU to the interior due to the excellent electrical conductivity of the MWCNT. Moreover, with the increasing content of MWCNTs, the electrical conductivity of MWCNT/TPU sharply increased, which led to strong charge migration. Based on the above characteristics, the short-circuit current of the TENG based on the MWCNT/TPU composite film improved by 100 times compared to the traditional dielectric TENG. Furthermore, even in high humidity environments, this TENG could maintain a stable output. Similarly, Wu *et al.* utilized the hysteretic and ordered charge drift behavior of dielectric polymers to construct TENGs with a stable and continuous (SC) output ability (SC-TENG).^87^ Under the action of the triboelectric electric field, the charge generated by triboelectrification migrates directionally from the surface to the inside of the polyurethane (PU) due to its high leakage property. Additionally, using a 1 mm-thick PU film ensures both excellent wear resistance and high output performance of the TENG.

In addition, charge migration can be used to achieve high-performance DC output. By coupling the charge migration and the ternary dielectric triboelectrification effect, Li *et al.* developed a DC-TENG with improved output performance, as shown in Fig. 11c.^48^ After continuous friction, a lot of positive and negative charges accumulated on the surfaces of the PA and PTFE films, respectively. The leakage current occurred between the electrode and PA film due to the high electric field, resulting in a DC output in the external circuit. The testing results show that the charge density output has a highly linear relationship with the leakage current of the positive tribo-layer. Moreover, the electronegativity of the triboelectric material is also vital for the charge density output. Therefore, they established the ternary dielectric evaluation rules for material selection and improving performance. After parameter optimization, the DC-TENG based on PA/PET/PTFE achieved an ultrahigh average power density of 6.15 W m^−2^ Hz^−1^. Similarly, Zhang *et al.* proposed a novel insulator-based quasi-tribovoltaic nanogenerator (I-Q-TVNG) with ultrahigh voltage and power by utilizing charge drift in the dielectric material.^152^ This I-Q-TVNG exhibits an ultrahigh output voltage of 2324 V and an average power of 11.8 mW. Additionally, it can maintain a nearly constant DC output with a crest factor of approximately 1.0204.

The detailed output performance of TENGs with the utilization of charge drift is shown in Table 6. Given the above, the triboelectric charges generated by triboelectrification can migrate from the surface of the material to the inside of the material through the selection of triboelectric materials with high leakage current, achieving utilization of charge drift and thus further output performance improvement.

**Table 6:** The summary of TENG with utilization of charge drift

Strategy	Triboelectric material	Output performance				Ref.
		*V*_OC_	*I*_SC_	*Q*_SC_	Output power	
Internal-space-charge zones	Au/PDMS	—	—	168 μC m^−2^	1 W m^−2^	145
Dielectric volume effect	Polyurethane foam film	9 kV	100 μA	2.8 μC	40.9 W m^−2^ Hz^−1^	46
Charge dispersion strategy	MWCNTs/TPU	320 V	400 μA	—	20 mW	47
Charge migration	Polyurethane	—	7.4 μA	879 nC	9.4 W m^−2^ Hz^−1^ (average)	87
Charge leakage effect and the ternary dielectric triboelectrification effect	PA/PTFE@PET (PET as the intermediate material)	3000 V	—	7.12 mC m^−2^	6.15 W m^−2^ Hz^−1^ (average)	48
Charge extraction	Nitrocellulose/PU	2324 V	1.7 mA m^−2^	—	11.8 mW (average)	152

## 6 Conclusion and future perspectives

In conclusion, this work outlined the recent research in the development of material design for improving TENG's output performance. Strategies discussed for high surface charge density include surface morphological modification, chemical modification, dielectric material doping, and charge injection. Furthermore, methods for improving the TENG's output by inhibiting air breakdown and utilizing charge drift have also been discussed. This paper is of great significance to the research of triboelectric materials, and also helpful to the practical research and innovation of TENGs. This review can significantly contribute to the research on triboelectric materials and advance practical studies of TENGs. The challenges and future perspectives for improving the output performance of TENGs have been summarized below.

### 6.1 Challenges

(1) The fundamental mechanisms of contact electrification still need further research. Several hypotheses of contact electrification have been proposed, but a definitive conclusion has not yet been established.

(2) The coupling mechanisms between the triboelectric effect and other effects, such as the piezoelectric effect, photovoltaic effect and magnetization effect, need to be further systematically and comprehensively understood.

(3) The study of the leakage current of triboelectric materials for high-performance TENGs is insufficient. Most of the past research proposed that the leakage current of the material limited its output performance. However, a recent study shows that proper leakage current is beneficial to the improvement of TENGs' output performance.

(4) The applications of TENGs in harsh environments. Triboelectric materials must be endowed with functional characteristics while maintaining their triboelectric properties to meet the diverse requirements of TENGs in different environments and then advance the commercial development of TENGs.

### 6.2 Future perspectives

Although progress has been achieved in enhancing the output performance of TENGs, more work is required to effectively address the remaining challenges. The strategies for improving the TENGs' output performance are prospected from the following aspects: the triboelectric material is the most important part of TENGs, which directly affects the output performance of TENGs. Additionally, the output performance of TENGs is affected by the environment, such as high humidity, high temperature, *etc.* Therefore, the ideal triboelectric material should have a high charge density, good mechanical stability, and environmental adaptability. New triboelectric materials need to be developed to obtain high-output performance in harsh environments. In high humidity environments, on the one hand, the properties of the triboelectric material can be changed by chemical modification so that it can maintain high output performance under high humidity conditions. On the other hand, it would also be a good choice to search for a material that can couple the triboelectric effect and the moisture-generating effect. At present, the most obvious technology to improve the TENG output performance is the power management system (PMS), which is one of the key technologies to realize the application of TENG technology. Some power management systems would provide a bias voltage to the friction material during operation. This bias voltage polarizes the triboelectric material, thus improving the output performance of TENGs. Composite films (especially those filled with high-dielectric nanoparticles) are also a promising research topic in this realm.

These important issues highlight great opportunities for researchers across different sectors to enhance the output performance of TENGs. The continuous technical innovation and theoretical research promote its application prospects in the field of energy harvesting and self-powered sensing, which is crucial for the widespread commercial applications of TENGs.

## Author contributions

X. Li: investigation, data curation, formal analysis, visualization, writing – original draft, editing. Q. Yang and D. Ren: investigation, review, editing. Q. Li, H. Yang, X. Zhang: review, editing. Y. Xi: resources, supervision, funding acquisition, writing – review & editing.

## Conflicts of interest

There are no conflicts to declare.
